# Lost but Not Forgotten—The Economics of Improving Patient Retention in AIDS Treatment Programs

**DOI:** 10.1371/journal.pmed.1000174

**Published:** 2009-10-27

**Authors:** Gregory P. Bisson, Jeffrey S. A. Stringer

**Affiliations:** 1 University of Pennsylvania School of Medicine, Department of Medicine, Division of Infectious Diseases, Philadelphia, Pennsylvania, United States of America; 2 University of Alabama School of Medicine, Department of Obstetrics and Gynecology, Division of International Women's Health, Birmingham, Alabama, United States of America; 3 Centre for Infectious Disease Research in Zambia, Lusaka, Zambia

## Abstract

Gregory Bisson and Jeffrey Stringer discuss the implications of a new study showing how loss to follow-up affects the effectiveness of a public sector HIV program in Côte d'Ivoire.

**Linked Research Article**

This Perspective discusses the following new study published in *PLoS Medicine*:

Losina E, Touré H, Uhler LM, Anglaret X, Paltiel D, et al. (2009) Cost-effectiveness of Preventing Loss to Follow-up in HIV Treatment Programs: a Côte d'Ivoire Appraisal. PLoS Med 6(10): e1000173. doi:10.1371/journal.pmed.1000173

## Loss to Follow-up after ART Initiation

The global HIV epidemic has been described as a public health emergency, which is both accurate and misleading. It is accurate because despite years of coordinated efforts, millions of people worldwide remain in urgent need of antiretroviral therapy (ART) [1]. But the term emergency is also misleading because it implies an acuity that, like a flood or famine, can be expected to abate once appropriate relief measures are delivered. Of course, the HIV pandemic is decades old, HIV care is life-long, and the HIV-infected population is young; the end of this public health emergency is nowhere in sight.

Tracking the progress of early ART scale-up efforts initially focused on tallying the number of individuals in need that actually started ART. A number of reports from Africa and other settings documented excellent early ART response rates, even in the face of severe resource constraints [2]–[4]. However, many of these reports also told another story. The introduction of AIDS care in many sites has represented a monumental shift in the way medical care is delivered—from a model of episodic treatment of symptomatic individuals with, for example, malaria, to one of life-long, chronic care. As such, the issue of patient attrition, never particularly considered or measured previously, emerged as a major issue. In a 2007 systematic review of patient retention in ART programs in sub-Saharan Africa, Rosen et al. found that only about 62% of those started on ART remained in care at 24 months after initiation [5]. Moreover, loss to follow-up (LTFU), a term used to describe patients who fail to present to clinic for a certain period of time and are not known to have died, accounted for most of the attrition.

Interruptions in ART are a problem from a patient care perspective because of the associated risk of incomplete virologic suppression [6], ongoing HIV transmission [7], inflammatory events, immunologic decline, opportunistic infections, and death [8]. LTFU is also particularly problematic from the standpoint of clinic efficiency because attempting to locate these patients after they become “late” to clinic is resource-intense and often unsuccessful. In Zambia's large national treatment program, for example, only one-third of patients who were late could be contacted, often after multiple attempts [9]. Finally, LTFU may lead not only to overly optimistic estimates of program response rates but to biased risk factors for death after ART initiation as well [10].

## A Cost-Effectiveness Study from Côte d'Ivoire

In the current issue of *PLoS Medicine*, Losina et al. show the dramatic contribution of LTFU to overall program effectiveness and cost-effectiveness of a public-sector program in Côte d'Ivoire [11]. Although data on the efficacy of interventions to reduce LTFU in African settings are scarce, hypothetical interventions can be created and their costs estimated. This approach has allowed Losina and colleagues to perform a series of threshold analyses, where interventions of known cost are evaluated over a range of effectiveness estimates. The resultant range of incremental cost-effectiveness ratios are plotted against consensus estimates of what the Ivoirian health sector would be willing to pay.

The authors used real-world cost and patient outcomes data from ACONDA, a local nongovernmental organization supporting ART delivery in Côte d'Ivoire to inform the well-validated Cost-Effectiveness of Preventing AIDS Complications (CEPAC) International simulation model. They evaluated the cost effectiveness of four hypothetical interventions to reduce LTFU, which included: (1) removing user co-payments from antiretroviral and (2) opportunistic infection drugs, (3) training providers in methods to improve patient retention, and (4) provision of meals and transport reimbursement for patient follow-up visits. The interventions were considered as if implemented incrementally (e.g., number 4 was not implemented without number 3.)

Among the most interesting findings of the analysis is its base case estimate of the contribution of LTFU to overall reductions in program effectiveness. In the 6,703-patient ACONDA-supported program, for instance, the authors estimate that LTFU results in more than 11,000 patient-years of lost life. It is in the context of this background that these hypothetical patient-retention interventions must be considered. For instance, removing user co-payment from the cost of antiretroviral drugs, an intervention estimated to cost $22 per patient per year, would only have to result in a 12% improvement in LTFU in order to meet the authors' criteria for cost-effectiveness in Côte d'Ivoire. On the other side of the spectrum, a combination of all four hypothetical interventions, estimated to cost $77 per patient per year, would need to be 41% effective to reach the cost-effectiveness threshold.

The reported estimates were most sensitive to modeled costs of second-line ART (which in Côte d'Ivoire contains much more expensive protease-inhibitor drugs). And while programs with the greatest LTFU rates stood the most to gain from the interventions, sensitivity analyses indicated that even programs with more modest loss rates might find such interventions to be cost-effective.

## The Global HIV Emergency: Acute ART Scale-up and Chronic HIV Care

Losina and colleagues' study signals an important widening of research attention to include not only the relative excitement of expanding ART coverage but also the less glorious work of improving chronic HIV care. The paper further quantifies the well-known problem of LTFU and provides a cost-effectiveness framework within which policy makers can begin to consider how to make their ART programs succeed over the long run. Although the efficacy of interventions to reduce LTFU remains largely unknown, this analysis makes clear that even interventions of modest efficacy are likely to fall well within the range of accepted cost-effectiveness. Clinical trials or other comparative studies aimed at evaluating such interventions would seem to be of high priority.

Given the financial scope of the global HIV/AIDS response, even small gains in program efficiencies and cost-effectiveness could translate into huge savings and, by extension, new lives saved. For this reason, the major AIDS donors, such as the US President's Emergency Plan For AIDS Relief (PEPFAR) and the Global Fund, should be keenly interested in this issue, and willing to invest in strategies to improve retention. Individual country programs also have a huge stake in minimizing LTFU. Not only do retained and appropriately treated patients have better clinical outcomes, they presumably are much less infectious and capable of transmitting the virus to others. Thus, treating and preventing HIV go hand in hand. Improving retention in HIV/AIDS care makes clear programmatic sense and, as Losina and colleagues demonstrate, it makes economic sense as well.

## Abbreviations

ART: antiretroviral therapy
LTFU: loss to follow-up
